# MicroRNA-485-5p inhibits glioblastoma progression by suppressing E2F transcription factor 1 under cisplatin treatment

**DOI:** 10.1080/21655979.2021.1982269

**Published:** 2021-11-02

**Authors:** Conggang Huang, Lan Ma, Faliang Duan, Ruixue Li, Yanguo Zhang, Yuan Wang, Ming Luo, Zhuqiang He, Zhihua Luo

**Affiliations:** a Department of Neurosurgery, The First Hospital of Wuhan, Wuhan, Hubei, China; b Department of Intensive Care Unit, The Sixth Hospital of Wuhan, Wuhan, Hubei, China

**Keywords:** Cisplatin, miR-485-5p, e2f1, glioblastoma, cell growth

## Abstract

Cisplatin (CDDP) has been widely used for glioblastoma treatment. miR-485-5p and E2F transcription factor 1 (E2F1) dysfunction has been reported in glioblastoma. Nonetheless, whether CDDP affects glioblastoma progression via the miR-485-5p-E2F1 axis requires investigation. The expression of miR-485-5p and E2F1 was investigated by quantitative real-time polymerase chain reaction or western blotting in glioblastoma tissues and cell lines. The interaction between miR-485-5p and E2F1 was confirmed using a luciferase assay. The malignancy of glioblastoma was detected using Cell Counting Kit-8, bromodeoxyuridine (BrdU), cell adhesion, flow cytometry, and transwell assays. We identified miR-485-5p downregulation and E2F1 upregulation in glioblastoma, and miR-485-5p inhibited cell growth and elevated cell apoptosis in glioblastoma cells after CDDP treatment. Moreover, miR-485-5p targeting E2F1 repressed cell growth and improved cell apoptosis in glioblastoma cells after CDDP treatment. Our study revealed that CDDP retarded glioblastoma cell development via the miR-485-5p-E2F1 axis, which may be a new direction for glioblastoma therapy.

## Introduction

Glioblastoma is the most common central nervous system malignancy with a poor prognosis [1,2]. Despite advances in understanding the molecular mechanisms of glioblastoma, patients still present a high recurrence rate and a low 5-year survival rate [3,4]. Thus, additional potential mechanisms underlying glioblastoma initiation and progression are crucial for glioblastoma diagnosis and treatment.

Cisplatin (CDDP) has been widely used as a cytotoxic drug in multiple tumors, including lung, bladder, and esophageal cancers [5–7]. CDDP treatment has been frequently applied in the diagnosis and treatment of glioblastoma [8]. For instance, Wang *et al*. [9] identified miR-152-3p as a CDDP sensitizer for glioblastoma in 2019. In 2020, Kim *et al*. [10] studied the effects of CDDP on cytotoxicity, apoptosis, autophagy, and the AKT/mTOR pathway in U87MG and U373MG cells. Therefore, we aimed to explore the mechanism of CDDP in glioblastoma, thereby improving the efficacy of CDDP in glioblastoma therapy.

MicroRNAs (miRNAs) consist of approximately 22 nucleotides, which bind to target gene mRNAs’ 3ʹ-untranslated regions (3ʹ-UTR) [11]. miRNAs have been reported to play important roles in regulating cell growth and differentiation in numerous cancers [12,13]. Evidence suggests that miRNAs are key regulators of glioblastoma development under CDDP treatment. For instance, miR-186 reversed CDDP resistance and inhibited Yin Yang 1 expression in glioblastoma, which significantly diminished glioblastoma proliferation [14]. Moreover, miR-152-3p coordinated with CDDP to repress SOS1 expression and reduce glioblastoma cell progression [9]. miR-485-5p has been identified as a suppressor in glioblastoma, hampering cell viability, migration, and invasion, while elevating cell apoptosis in glioblastoma U87 and U251 cells [15,16]. However, whether miR-485-5p influences glioblastoma progression under CDDP treatment remains unknown.

The E2F transcription factor 1 (E2F1) gene is located on chromosome 20q11.22 and consists of seven exons. It encodes a member of the E2F family of transcription factors, which plays a crucial role in controlling the cell cycle and regulating tumor suppressor proteins [17]. Evidence displays that E2F1 aggravates cancer progression as an oncogene [18]. E2F1 has been reported to facilitate glioblastoma development, where it accelerates cell growth under CDDP treatment [19]. One study revealed that miR-136 remarkably repressed E2F1 expression to promote CDDP chemosensitivity in glioma cells [20]. Notably, the function of the miR-485-5p-E2F1 axis in glioblastoma with CDDP treatment is unclear.

Therefore, we aimed to investigate the effect of the miR-485-5p-E2F1 axis in glioblastoma cells under CDDP treatment. It was hypothesized that CDDP-assisted miR-485-5p might reduce E2F1 expression following CDDP treatment in glioblastoma. This study provides novel insights into the molecular mechanisms underlying glioblastoma.

## Materials and methods

### Clinical Tissues, Cell Culture, and Cell Transfection

Human glioblastoma tissues and adjacent normal tissues were collected from 34 patients in our hospital with informed consent and approved by the Ethics Committee of our hospital. The patients’ baseline data are presented in Table 1. Human glioblastoma U251 and U87 cells were obtained from ATCC (VA, USA) and were maintained in Dulbecco’s Modified Eagle’s Medium (Gibco, TX, USA) supplemented with 10% fetal bovine serum (FBS; Gibco) at 37°C and 5% CO_2_. For CDDP or temozolomide (TMZ) treatment, CDDP (Cat#: 232,120, Sigma-Aldrich, MO, USA) or TMZ (Cat#: 34,219, Sigma-Aldrich) was dissolved in phosphate-buffered saline (PBS), and final concentrations of 10, 20, and 30 μM were added to the medium. The miR-485-5p inhibitor, mimic, E2F1 overexpression plasmid (OE), and their negative controls (NC) were obtained from GenePharm (Shanghai, China). When cells reached 50% confluence, U251 and U87 cells were transfected using Lipofectamine 3000 (Invitrogen, MA, USA), and the following experiments were conducted after 48 h of transfection.

**Table 1.** The baseline characteristics of 34 glioblastoma patients

Categories	Patients (Total n = 34)
Age (year)	
<60	19 (55.88%)
≥60	15 (44.12%)
Gender	
Male	16 (47.06%)
Female	18 (52.94%)
Karnofsky score	
≤70 score	21 (61.76%)
≥80 score	13 (38.24%)
Tumor location	
Fronto lobe or temporal lobe	11 (32.35%)
Other lobe	23 (67.65%)
Recurrence	
Yes	15 (44.12%)
No	19 (55.88%)
MGMT methylight	
methylated	18 (52.94%)
unmethylated	16 (47.06%)
IDH1 mutant	
mutated	5 (14.71%)
unmutated	29 (95.29%)
Smoking	
Yes	10 (29.41%)
No	24 (70.59%)
Family history of cancer	
Yes	21 (61.76%)
No	13 (38.24%)

### RNA Extraction and quantitative real-time polymerase chain reaction (qRT-PCR)

TRIzol reagent (Invitrogen) was used for RNA isolation, and the miRNeasy Mini Kit (Cat#: 217,004, QIAGEN, Hilden, Germany) was used for miRNA isolation from U251 and U87 cells. Next, cDNA transcribed from RNA was applied using the PrimeScript First Strand cDNA Synthesis Kit (Takara Bio, Shiga, Japan), and cDNA transcribed from miRNA was obtained using a miScript II RT Kit (Cat#: 218,161, QIAGEN). qRT-PCR was performed using SYBR Premix Ex Taq (Takara Bio) for E2F1 expression and the miScript SYBR Green PCR Kit (Cat#: #218,075, QIAGEN) for miR-485-5p expression. Relative expression was normalized to Glyceraldehyde-3-phosphate dehydrogenase (GAPDH) or small RNA U6 (U6) using the 2-^ΔΔCt^ method [21]. All primer sequences are listed in Table 2.

**Table 2.** Primer sequences

Genes	Primer sequences
miR-485-5p	Forward:5ʹ-AGAGGCTGGCCGTGAT-3ʹ Reverse:5ʹ-ATGTGTTGCTGTGTTTTGTCG-3’
E2F1	Forward:5ʹ-CTGCAGCAACTGCAGGAGAG-3ʹ Reverse:5ʹ-CTCCGAAAGCAGTTGCAGC-3’
GAPDH	Forward:5ʹ-TCCTCTGACTTCAACAGCGACAC-3ʹ Reverse:5ʹ-CACCCTGTTGCTGTAGCCAAATTC-3’
U6	Forward:5ʹ-AACGAGACGACGACAGAC-3ʹ Reverse:5ʹ-GCAAATTCGTGAAGCGTTCCATA-3’

### Cell counting kit-8 (CCK8) assay

U251 and U87 cells were seeded at a density of 1 × 10^4^ cells/well in 96-well plates. After adherence, the cells were treated with different concentrations of CDDP or TMZ for 48 h, followed by the incubation with 10 μL CCK8 solution (Dojindo, Kumamoto, Japan) for 2 h. Furthermore, a different set of cells was treated with 10 μM CDDP and incubated with 10 μL CCK8 solution for 2 h at four time points (0, 24, 48, and 72 h). The OD value at 450 nm was detected using a multimode plate reader (Thermo Fisher Scientific, MA, USA) [20,22].

### Bromodeoxyuridine (BrdU) assay

The BrdU assay was performed according to a previous study [23]. U251 and U87 cells were seeded at a density of 1 × 10^4^ cells/well in 96-well plates. After cells reached 80% confluence, the cells were labeled with BrdU (Cat#: ab126556, Abcam, Cambridge, UK) for 12 h. After washing twice with PBS, the cells were permeabilized and incubated with BrdU antibody for 2 h at room temperature. Then, the cells were incubated with anti-mouse antibody (Abcam, Cambridge, UK) for 1 h and subjected to a multimode-plate-reader at OD 450 nm (Thermo Fisher Scientific).

### Cell adhesion assay

Collagen I solution (Sigma-Aldrich) was added to a 96-well plate for cell adhesion detection, and 2 × 10^4^ U251 and U87 cells were cultured into the plate at 37°C for 4 h. Post culture, the medium was discarded, and the cells were treated with 3-(4,5-dimethylthiazol-2-yl)-2,5-diphenyltetrazolium bromide reagent (C0009S, Beyotime, Jiangsu, China) for 2 h at 37°C. Then, 100 µL dimethyl sulfoxide was added to each well, and the OD value at 570 nm was detected using a multimode-plate-reader (Thermo Fisher Scientific) [24].

### Cell apoptosis assay

As previously described [25], the Annexin V-FITC Apoptosis Detection Kit (Cat#: 556,547; BD, NJ, USA) was used to detect apoptosis in U251 and U87 cells. Cells were harvested and treated with 5 µL fluorescein isothiocyanate and propidium iodide. After incubation for 20 min in the dark, the cells were washed twice, suspended, and analyzed by flow cytometry. The sum of the two right quadrants represented cell apoptosis, which was calculated using the FlowJo software (Tree Star, OR, USA).

### Transwell assay

U251 and U87 cell migration was measured using a transwell chamber (Cat#: 3244, Corning, NY, USA) in a 24-well plate. The bottom compartment was prepared with 10% FBS medium, and cell suspensions (2 × 10^5^ cells) without FBS were added to the top compartment. After 24 h of incubation, cells in the upper layer were removed, and cells in the lower layer were treated with 4% paraformaldehyde and 0.5% crystal violet for 15 min. Finally, the migrated cells were randomly captured using a microscope (Olympus, Tokyo, Japan) [26].

### Luciferase reporter assay

This assay was performed according to a previous study [27]. Wild-type (WT) sequence of E2F1 3ʹ-UTR based on the binding site, and a random mutated (MUT) sequence was constructed into the pmiRGLO vectors. U251 and U87 cells were co-transfected with pmiRGLO-E2F1-WT or pmiRGLO-E2F1-MUT and either miR-NC or miR-485-5p using Lipofectamine 3000. After 72 h, Firefly and Renilla luciferase activities were applied to the Luciferase Assay Kit (Abcam). Renilla luciferase activity was used as an internal control.

### Western blot

E2F1 protein expression was measured by western blotting, as previously described [28]. The protein from the cells was extracted using a cell lysis buffer. Then, 20 µg protein was separated using 10% sodium dodecyl sulfate polyacrylamide gel electrophoresis, transferred onto a polyvinylidene fluoride membrane, and blocked with 5% nonfat milk. The E2F1 (1:1000, Cat#: ab112580, Abcam) and GAPDH (1:5000, Cat#: 5174, CST, MA, USA) antibodies were used to incubate the membranes overnight at 4°C. The membranes were then washed and incubated with anti-HRP-Rabbit antibody (1:5000, Cat#: 80,403, CST) for 1 h at 25°C. Finally, protein bands were detected using enhanced chemiluminescence (ECL) reagents (Thermo Fisher Scientific, MA, USA).

## Statistical analysis

Independent sample *t*-tests for two-group comparisons and one-way analysis of variance with Dunnett’s post hoc test for multiple group comparisons were used for data analysis, which was performed using GraphPad (CA, USA). The data are presented as the mean ± standard deviation based on three independent experiments. Pearson analysis was used to determine the correlation between E2F1 expression and miR-485-5p expression in glioblastoma tissues. Statistical significance was set at *P* < 0.05.

## Results

In this study, we hypothesized that miR-485-5p inhibits glioblastoma progression by suppressing E2F1 expression under CDDP treatment. First, we identified the key miRNA (miR-485-5p) and target gene (E2F1) through bioinformatics analysis and found that CDDP treatment upregulated miR-485-5p levels in glioma cells and downregulated E2F1 levels. To investigate the role of CDDP, miR-485-5p, or E2F1 in glioma cells, we evaluated the effects of CDDP combined with miR-485-5p or E2F1 expression on the viability, proliferation, apoptosis, and invasion of glioma cells. Additionally, we validated target binding between miR-485-5p and E2F1. It was revealed that the CDDP-miR-485-5p-E2F1 axis may be a new direction for glioblastoma therapy.

## Upregulated E2F1 level and downregulated miR-485-5p level were observed in glioblastoma

**Figure 1.**
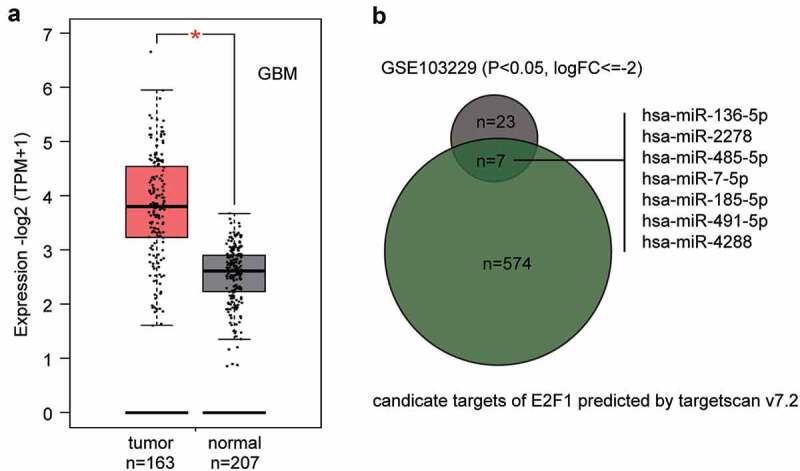
The identification of miR-485-5p as the miRNA of interest in this study.(a) The expression level of E2F1 in GBM (glioblastoma multiforme) from GEPIA database (http://gepia2.cancer-pku.cn/). (b) The intersection of the predicted target miRNAs of E2F1 by targetscan v7.2 and the significantly downregulated miRNAs from GSE103229 data series. FC: fold change

The high E2F1 expression in GEPIA glioblastoma data is shown in Figure 1a. GSE103229 from GEO DataSets stored the miRNAs expression profile in glioblastoma samples and normal brain samples. According to the ceRNA mechanism, there is a negative correlation between miRNA and its target genes. Therefore, to identify a potential upstream regulator miRNA of E2F1 in glioblastoma, we intersected the significantly downregulated miRNAs from the GSE103229 data series (screening criteria: P < 0.05 and logFC≤-2) and the predicted miRNAs that could regulate E2F1 by TargetScan algorithm. Seven candidates were identified to target E2F1 and be downregulated in glioblastoma samples (Figure 1b). The candidates are ranked according to their downregulation levels in GSE103229. miR-136-5p has been reported to be a significant tumor suppressor in glioblastoma and is the most significantly downregulated miRNA among the seven; however, it has been previously studied in CDDP resistance in glioblastoma [20]. The second most ranked miRNA, miR-485-5p, is a significant tumor suppressor in glioblastoma; however, it has never been studied in glioblastoma chemoresistance. Thus, we chose miR-485-5p as our study focus.

## CDDP inhibited glioblastoma progression by upregulating miR-485-5p

**Figure 2.**
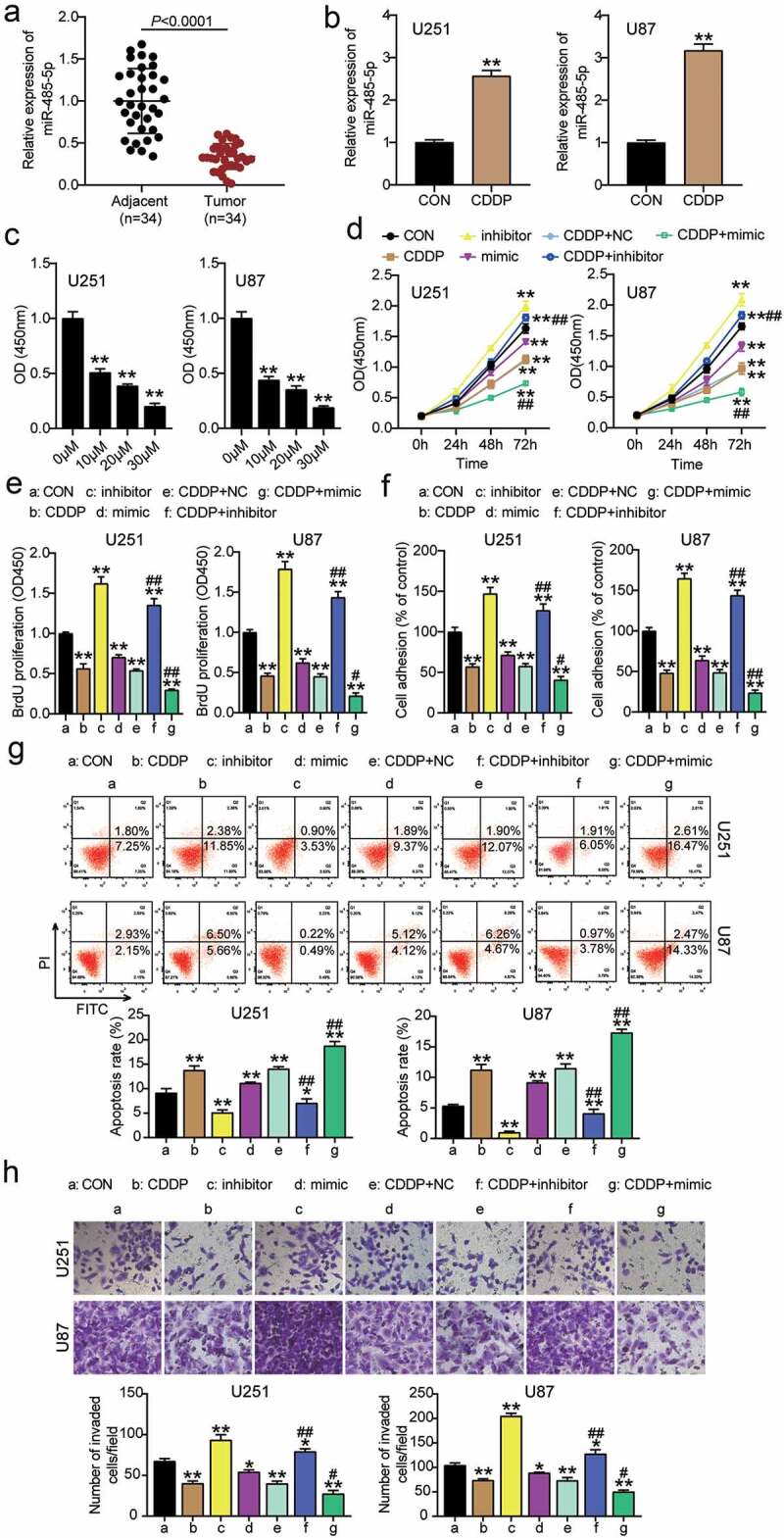
CDDP inhibited cell growth and promoted apoptosis by upregulating miR-485-5p levels in glioblastoma. The miR-485-5p level was detected in glioblastoma tissues and adjacent normal tissues by qRT-PCR. (b) The miR-485-5p level was detected in U251 and U87 cells treated with CDDP by qRT-PCR. **, *P* < 0.001 compared with CON. (c) Cell viability was detected in U251 and U87 cells treated with different concentration of CDDP at 0, 10, 20,30 μM by CCK8 assay. **, *P* < 0.001 compared with 0 μM. (d) Cell viability was detected in U251 and U87 cells transfected with NC, inhibitor, mimic and treated with CDDP by CCK8 assay. (e) Cell proliferation was detected in U251 and U87 cells transfected with NC, inhibitor, mimic and treated with CDDP by BrdU assay. (f) Cell adhesion was detected in U251 and U87 cells transfected with NC, inhibitor, mimic and treated with CDDP by MTT regent. (g) Cell apoptosis was determined in U251 and U87 cells transfected with NC, inhibitor, mimic and treated with CDDP by FITC apoptosis detection kit. (h) Cell migration was detected in U251 and U87 cells transfected with NC, inhibitor, mimic and treated with CDDP by transwell assay. *, *P* < 0.05; **, *P* < 0.001 compared with CON. ^#^, *P* < 0.05; ^##^, *P* < 0.001 compared with CDDP+NC. CON, blank control; inhibitor, miR-485-5p inhibitor; mimic, miR-485-5p mimic; CDDP+NC, CDDP+ negative control; CDDP+ mimic, CDDP+ miR-485-5p mimic; CDDP+ inhibitor, CDDP+ miR-485-5p inhibitor

To investigate the function of CDDP and miR-485-5p in glioblastoma, miR-485-5p expression in glioblastoma tissues was examined. The results showed that miR-485-5p expression was downregulated in glioblastoma tissues (Figure 2a). When CDDP was used to treat U251 and U87 cells, miR-485-5p expression increased (Figure 2b). Subsequently, we determined cell viability in U251 and U87 glioblastoma cells treated with CDDP at concentrations of 0, 10, 20,30 μM, and found that cell viability was significantly decreased at 10 μM treatment, which was the half maximal inhibitory concentration (IC50) (Figure 2c). Therefore, we selected 10 μM CDDP for subsequent experiments. In addition, to confirm the CDDP efficacy in glioma cells, cells were also targeted with TMZ. The results showed that cell viability decreased with an increase in TMZ concentration. The OD value of TMZ-treated cells was similar to that of CDDP-treated cells (Supplementary Figure 1). After transfection of the miR-485-5p inhibitor or miR-485-5p mimic in U251 and U87 cells with CDDP treatment, we found that the miR-485-5p inhibitor increased cell viability, miR-485-5p mimic decreased cell viability, and treatment with an inhibitor or mimic accentuated or reversed the inhibitory effect of CDDP on cell viability (Figure 2d). miR-485-5p inhibitor elevated cell proliferation, while the CDDP and miR-485-5p mimic groups reduced cell proliferation (Figure 2e). In addition, the enhanced or decreased cell adhesion of miR-485-5p inhibitor or miR-485-5p mimic were all partially eliminated or sharpened by the inhibitory adhesion of CDDP (Figure 2f). Moreover, 50% reduced cell apoptosis in the miR-485-5p inhibitor group was observed, but cell apoptosis observed in the CDDP or miR-485-5p mimic groups was higher (Figure 2g). Simultaneously, apoptosis changes caused by transfection with mimic or inhibitor were observed to be reversed or aggravated by CDDP treatment (Figure 2g). Finally, cell migration was elevated in the miR-485-5p inhibitor, while the CDDP or miR-485-5p mimic groups repressed cell migration (Figure 2h). Altogether, these data suggest that CDDP suppresses cell growth and enhances cell apoptosis by promoting miR-485-5p in glioblastoma.

## E2F1 is a target of miR-485-5p in glioblastoma

**Figure 3.**
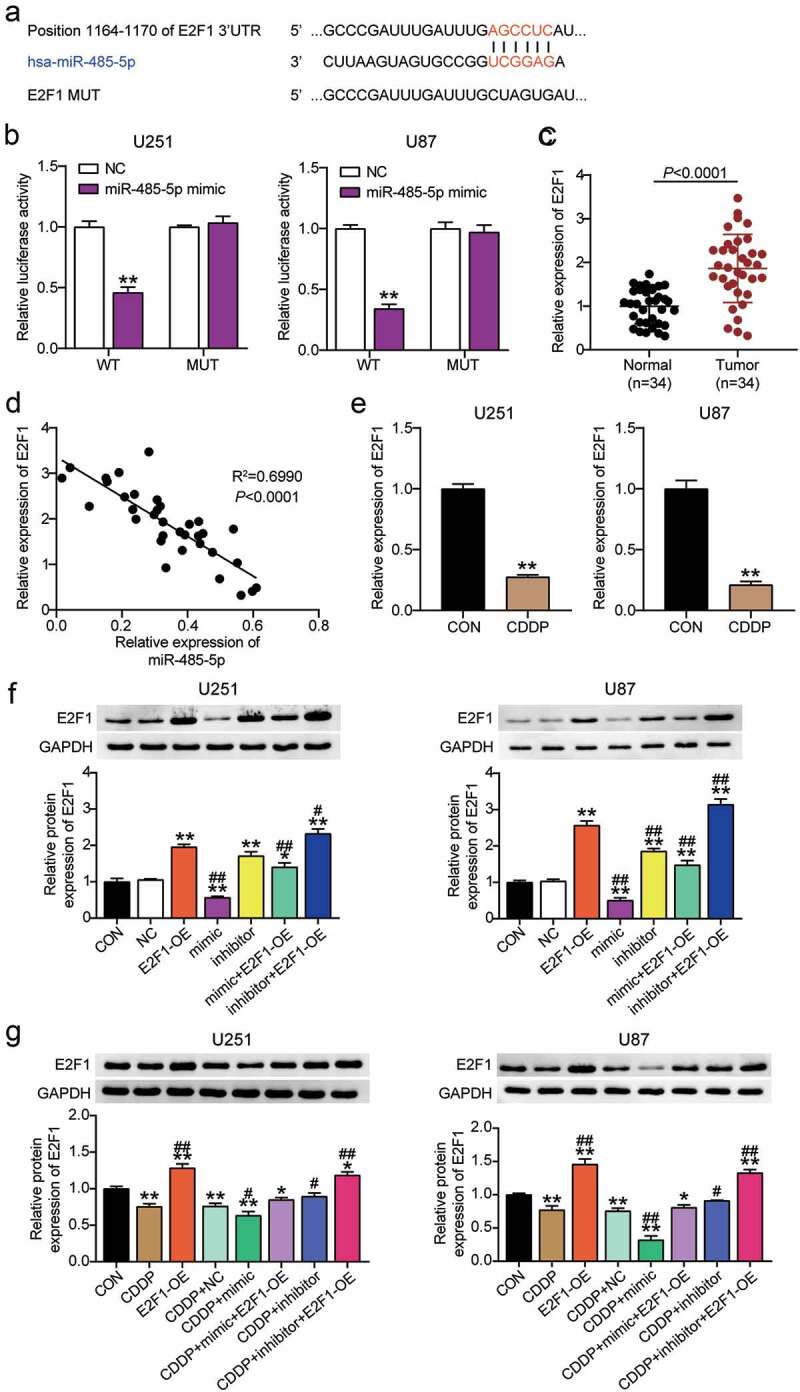
E2F1 was a target of miR-485-5p in glioblastoma. TargetScan showed the predicted binding sequences of E2F1 3ʹ-UTR for miR-485-5p. (b) Dual luciferase assay was performed in cells co-transfected with plasmids E2F1 -WT or E2F1 -MUT and miR-NC or miR-485-5p mimic in U251 and U87 cells. **, *P* < 0.001 compared with NC. (c) The E2F1 expression was detected in glioblastoma tissues and adjacent normal tissues by qRT-PCR. (d) The correlation between E2F1 expression and miR-485-5p level was analyzed by Pearson analysis. (e) The E2F1 expression was detected in U251 and U87 cells treated with CDDP by qRT-PCR. **, *P* < 0.001 compared with CON. (f) The E2F1 protein expression was detected in U251 and U87 cells transfected with E2F1-OE, NC, mimic, inhibitor, mimic+ OE and inhibitor+ OE by WB detection. (g) The E2F1 protein expression was detected in U251 and U87 cells transfected with E2F1-OE, NC, mimic, inhibitor, mimic+ OE, inhibitor+ OE, and treated with CDDP by WB detection. *, *P* < 0.05; **, *P* < 0.001 compared with CON. ^#^, *P* < 0.05; ^##^, *P* < 0.001 compared with CDDP+NC. CON, blank control; E2F1-OE, E2F1-overexpression; CDDP+NC, CDDP+ negative control; CDDP+ mimic, CDDP+miR-485-5p mimic; CDDP+ inhibitor, CDDP+ miR-485-5p inhibitor; CDDP+ mimic+ OE, CDDP+ miR-485-5p mimic+E2F1-overexpression; CDDP+ inhibitor+ OE, CDDP+ miR-485-5p inhibitor+E2F1-overexpression

To determine whether E2F1 interacted with miR-485-5p, the binding site between E2F1 and miR-485-5p was obtained using TargetScan Human 7.2 (Figure 3a). The luciferase activity in cells treated with pmiRGLO-E2F1 E2F1 3-UTR WT and miR-485-5p mimic was decreased; however, no difference was observed in cells transfected with pmiRGLO-E2F1 3ʹUTR MUT, suggesting that miR-485-5p interacted with E2F1 in U251 and U87 cells (Figure 3b). Next, we found that E2F1 expression was upregulated in glioblastoma tissues from 34 patients (Figure 3c), and a negative correlation between E2F1 expression and miR-485-5p expression was observed in glioblastoma tissues from 34 patients (Figure 3d). E2F1 expression was downregulated in U251 and U87 cells following CDDP treatment (Figure 3e). In addition, compared with the control group, the E2F1 protein level was upregulated in the E2F1-OE and miR-485-5p inhibitor groups, and downregulated in the mimic group; CDDP treatment facilitated the effect of miR-485-5p mimic and offset the effect of the inhibitor, suggesting that miR-485-5p is upstream of E2F1 under CDDP treatment in glioblastoma (Figure 3f and g).

## CDDP hampered cell progression via the miR-485-5p-E2F1 axis in glioblastoma

**Figure 4.**
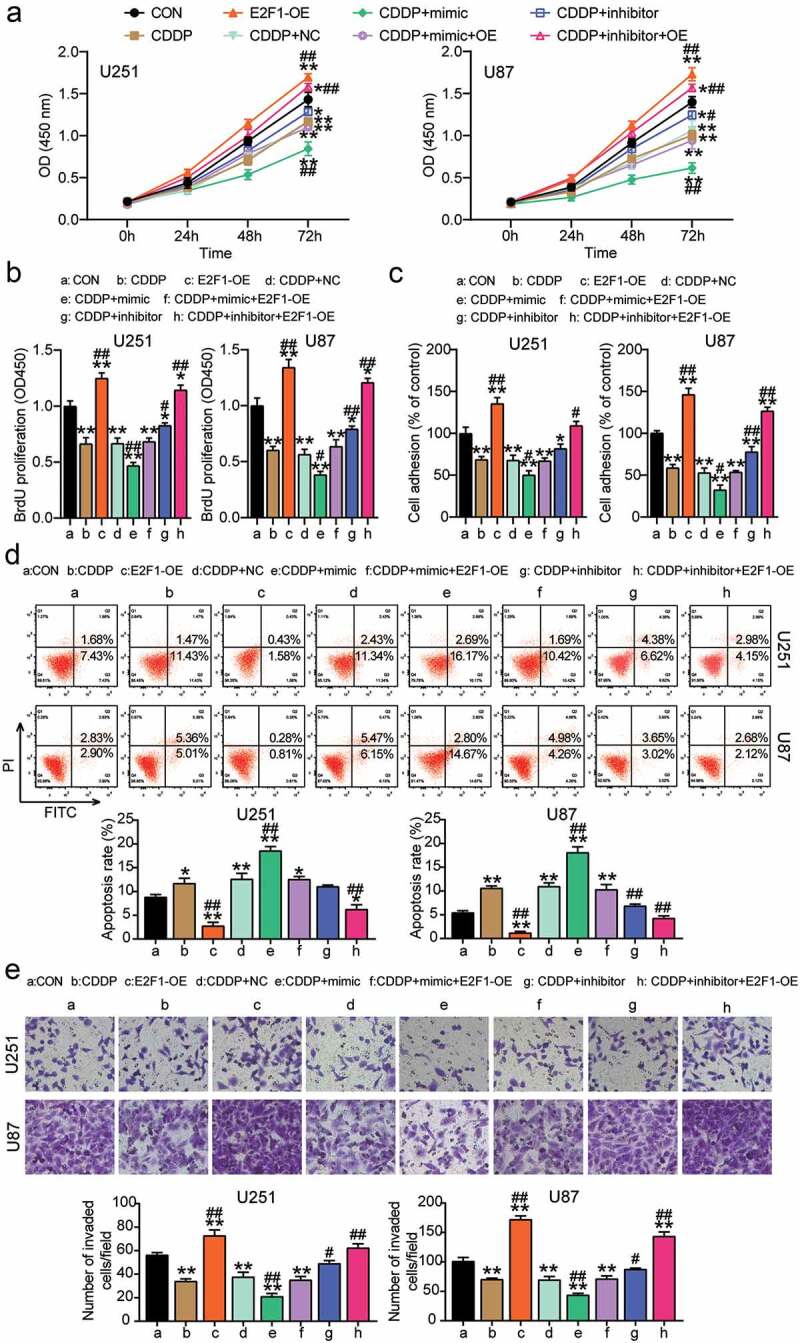
CDDP inhibited cell growth and promoted apoptosis via miR-485-5p-E2F1 axis in glioblastoma. Cell viability was detected in U251 and U87 cells transfected with E2F1-OE, NC, mimic, inhibitor, mimic+ OE, inhibitor+ OE, and treated with CDDP by CCK8 assay. (b) Cell proliferation was detected in U251 and U87 cells transfected with E2F1-OE, NC, mimic, inhibitor, mimic+ OE, inhibitor+ OE, and treated with CDDP by BrdU assay. (c) Cell adhesion was detected in U251 and U87 cells transfected with E2F1-OE, NC, mimic, inhibitor, mimic+ OE, inhibitor+ OE, and treated with CDDP by MTT regent. (d) Cell apoptosis was determined in U251 and U87 cells transfected with E2F1-OE, NC, mimic, inhibitor, mimic+ OE, inhibitor+ OE, and treated with CDDP by FITC apoptosis detection kit. (e) Cell migration was detected in U251 and U87 cells transfected with E2F1-OE, NC, mimic, inhibitor, mimic+ OE, inhibitor+ OE, and treated with CDDP by transwell assay. *, *P* < 0.05; **, *P* < 0.001 compared with CON. ^#^, *P* < 0.05; ^##^, *P* < 0.001 compared with CDDP+NC. blank control; E2F1-OE, E2F1-overexpression; CDDP+ NC, CDDP+ negative control; CDDP+ mimic, CDDP+ miR-485-5p mimic; CDDP+ inhibitor, CDDP+ miR-485-5p inhibitor; CDDP+ mimic+ OE, CDDP+ miR-485-5p mimic+E2F1-overexpression; CDDP+ inhibitor+ OE, CDDP+ miR-485-5p inhibitor+E2F1-overexpression

To clarify whether the miR-485-5p-E2F1 axis participates in glioblastoma with CDDP treatment, the CCK8 assay was performed; it was observed that E2F1-OE upregulated cell viability, while this effect was reversed by CDDP and miR-485-5p mimic and aggravated by inhibitor (Figure 4a). Furthermore, E2F1-OE enhanced cell proliferation, while the effect was inhibited by CDDP and miR-485-5p mimic and promoted by the inhibitor (Figure 4b). Moreover, E2F1-OE promoted cell adhesion, while this effect was inhibited by CDDP and the miR-485-5p mimic and accelerated by the inhibitor (Figure 4c). Furthermore, E2F1-OE reduced cell apoptosis, while this effect was suppressed by CDDP and the miR-485-5p mimic and facilitated by the inhibitor (Figure 4d). Finally, E2F1-OE enhanced cell migration, but, this effect was hampered by CDDP and miR-485-5p mimics and aggravated by the inhibitor (Figure 4e). Collectively, the data indicate that CDDP hampers cell growth and promotes cell apoptosis via the miR-485-5p-E2F1 axis in glioblastoma.

## Discussion

This study revealed miR-485-5p downregulation and E2F1 upregulation in glioblastoma tissues. miR-485-5p hampered cell proliferation, adhesion, and migration, and elevated cell apoptosis in glioblastoma cells following CDDP treatment. Moreover, miR-485-5p targeting E2F1 repressed glioblastoma progression following CDDP treatment.

miRNAs have been shown to play pivotal roles in cancer with CDDP treatment. Liu *et al*. found that the knockdown of miR-6727-5 promoted the sensitivity of cervical cancer cells to CDDP [27]. Chen *et al*. showed that CDDP inhibited miR-132 expression in oral squamous cell carcinoma, thereby regulating the proliferation, invasion, and migration of cancer cells [26]. Moreover. Yang *et al*. reported that miR-29a significantly repressed prominin-1 (CD133) expression and contributed to CDDP resistance in CD133^+^ glioblastoma stem cells [29]. Guo *et al*. reported that Let-7b downregulation elevated cyclin D1 expression under CDDP treatment in glioblastoma cells [30]. Wang *et al*. found that 10 μM CDDP was an effective concentration for glioblastoma cell treatment, and miR-152-3p downregulation clearly decreased CDDP sensitivity [9]. Similarly, we found that 10 μM CDDP was an effective concentration for glioblastoma cell treatment. We further demonstrated that miR-485-5p retarded cell proliferation, adhesion, and migration, and elevated cell apoptosis in glioblastoma cells after CDDP treatment.

To date, E2F1-targeting treatment has been used in chemotherapy for colorectal and breast cancers [31,32]. One study reported that E2F1 overexpression in glioma-derived cell lines induced p53-independent apoptosis, which was further enhanced by ionizing radiation [33]. Another study showed that adenovirus-mediated transfer of E2F-1 potentiated the chemosensitivity of human glioma cells to TMZ and BCNU [34]. Importantly, Chen *et al*. revealed that silencing E2F1 enhanced tumor-suppressive functions, while E2F1 upregulation exhibited the opposite effect in glioma cells with CDDP treatment [20]. Consistent with this study, we demonstrated that E2F1 overexpression promoted glioblastoma cell proliferation, adhesion, and migration, and retarded cell apoptosis with CDDP treatment. Importantly, miR-485-5p could repress E2F1 expression and suppressed glioblastoma cell development under CDDP treatment. Our study is the first to show that the CDDP-miR-485-5p-E2F1 axis plays a key role in glioblastoma.

Although our study has revealed that miR-485-5p inhibits glioblastoma cell progression under CDDP treatment by targeting E2F1, the specific signaling pathways require further exploration. Meanwhile, animal models should be applied to confirm the role of the CDDP-miR-485-5p-E2F1 axis in glioblastoma.

## Conclusion

Taken together, our study revealed that repressed cell growth and elevated cell apoptosis of glioblastoma cells occurred via the miR-485-5p-E2F1 axis under CDDP treatment. Thus, the CDDP-miR-485-5p-E2F1 axis may be an innovative direction for glioblastoma therapy.

## Supplementary Material

Supplemental Material

## Data Availability

The datasets used and/or analyzed during the current study are available from the corresponding author on reasonable request.
