# Supplementary figures and images for: MicroRNA-485-5p inhibits glioblastoma progression by suppressing E2F transcription factor 1 under cisplatin treatment
Source: Bioengineered. 2021 Nov 2;12(1):8020–30. doi: 10.1080/21655979.2021.1982269 (PMC8806419; doi:10.1080/21655979.2021.1982269)

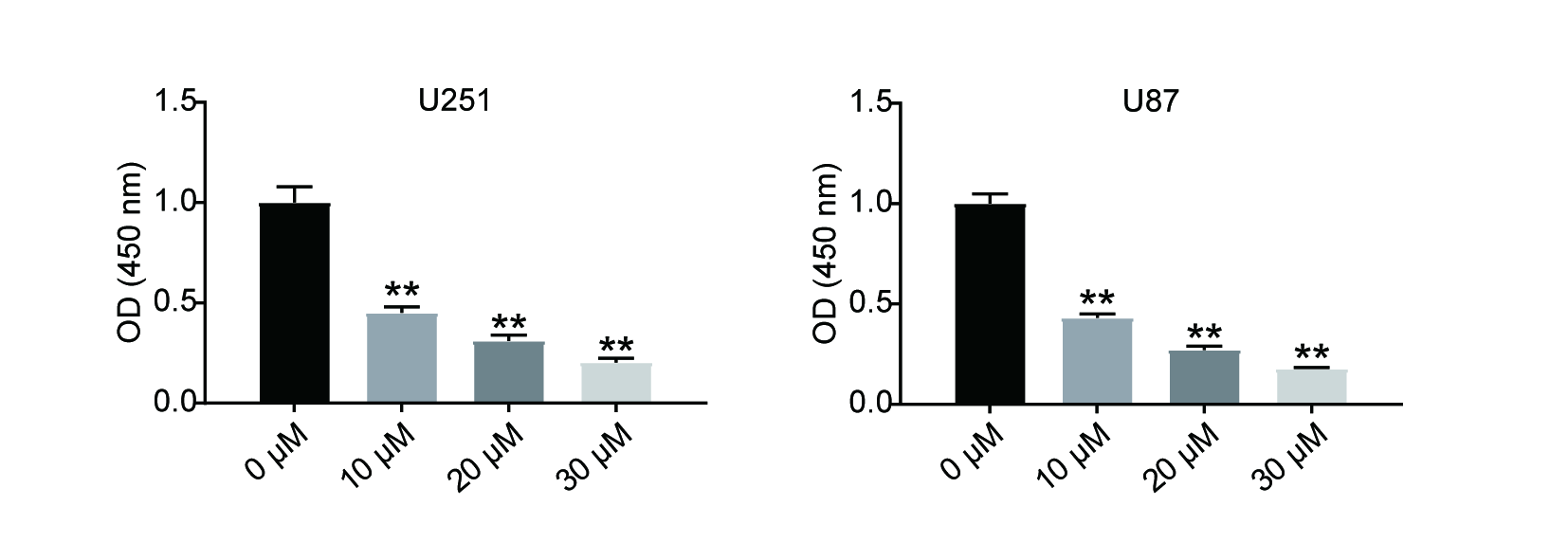

Supplement: Supplemental Material [file KBIE_A_1982269_SM8616.tif]
